# Potential of Novel Sequence Type of *Burkholderia* *cenocepacia* for Biological Control of Root Rot of Maize (*Zea* *mays* L.) Caused by *Fusarium temperatum*

**DOI:** 10.3390/ijms20051005

**Published:** 2019-02-26

**Authors:** Setu Bazie Tagele, Sang Woo Kim, Hyun Gu Lee, Youn Su Lee

**Affiliations:** Department of Applied Plant Sciences, Kangwon National University, Chuncheon 24341, Korea; setubazie@gmail.com (S.B.T.); ksw80@kangwon.ac.kr (S.W.K.); mye1991@kangwon.ac.kr (H.G.L.)

**Keywords:** *Burkholderia cenocepacia*, *Burkholderia contaminans*, *Fusarium temperatum*, MLSA-MLST, sequence type (ST), root rot, *Zea mays*

## Abstract

In this study, two *Burkholderia* strains, strain KNU17BI2 and strain KNU17BI3, were isolated from maize rhizospheric soil, South Korea. The 16S rRNA gene and multilocus sequence analysis and typing (MLSA-MLST) were used for the identification of the studied strains. Strain KNU17BI2, which belonged to *Burkholderia cenocepacia,* was of a novel sequence type (ST) designated ST-1538, while strain KNU17BI3 had a similar allelic profile with the seven loci of *Burkholderia contaminans* strain LMG 23361. The strains were evaluated in vitro for their specific plant growth promoting (PGP) traits, such as zinc solubilization, phosphate solubilization, ammonia production, 1-aminocyclopropane-1-carboxylate (ACC) deaminase activity, indole acetic acid (IAA) production, siderophore, and hydrolytic enzyme activity. Interestingly, the strains exhibited a positive effect on all of the tested parameters. The strains also showed broad-spectrum antifungal activity against economically important phytopathogens in the dual culture assay. Furthermore, the strains were evaluated under greenhouse conditions for their in vivo effect to promote plant growth and to suppress the root rot of maize that is caused by *Fusarium temperatum* on four Korean maize cultivars. The results of the greenhouse study revealed that both of the strains were promising to significantly suppress *fusarium* root rot and enhance plant growth promotion on the four maize cultivars. This study, for the first time, reported in vitro antifungal potential of *B. cenocepacia* of novel ST against economically important plant pathogens viz., *F. temperatum*, *Fusarium graminearum*, *Fusarium moniliforme*, *Fusarium oxysporum* f.sp. *melonis*, *Fusarium* subglutinans, *Phytophthora* *drechsleri,* and *Stemphylium lycopersici*. This is also the first report of zinc solubilization by *B. cenocepacia*. Moreover, the present research work reports, for the first time, about the potential of *B. cenocepacia* and *B. contaminans* to control the root rot of maize that is caused by *F. temperatum*. Therefore, we recommend further studies to precisely identify the bioactive chemical compounds behind such activities that would be novel sources of natural products for biological control and plant growth promotion of different crops.

## 1. Introduction 

Globally, *Fusarium* species cause several diseases of maize (*Zea mays* L.), such as seed rot, seedling blight, and stalk rot [1,2]. *Fusarium temperatum* has been reported to cause seed rot, seedling blight, and stalk rot of maize in different parts of the world [3,4], and it is necessary in the proper monitoring and control of disease in maize-growing areas [5].

Management of *Fusarium* root rot with chemicals has previously been reported [6,7]. However, due to environmental pollution and public concern, interest in the use of synthetic chemical fertilizers and pesticides has been exceedingly diminished [8]. Interestingly, the use of plant growth promoting rhizobacteria (PGPR), which plays a pivotal role in nutrient management, plant growth promotion, and disease management, has been increased [9]. Nevertheless, there is no information regarding the management of maize root rot that is caused by *F. temperatum*. 

Members of the genus *Burkholderia* are the highly adaptable micro-organisms that can inhabit highly diverse ecological niches [10]. Interestingly, the *Burkholderia* species are able produce plenty of secondary metabolites, which are novel sources of bioactive compounds [11] with proteolytic [12] and lipolytic activities [13]. Furthermore, rhizospheric *Burkholderia* species have been reported to solubilize the phosphorus and zinc from insoluble sources [14,15]. More recently, several studies suggested the potential biotechnological application of *Burkholderia* species to promote plant growth and control plant diseases [16,17,18].

The genus *Burkholderia* was initially defined by Yabuuchi et al. [19] to accommodate seven species of the former rRNA group II pseudomonads. Currently, the genus *Burkholderia* comprises more than 100 species [20]. However, *Burkholderia cepacia* complex (Bcc) bacteria are highly closely related species, in which the similarity level of the 16S rRNA gene sequence is more than 97.5% [18]. Interestingly, the multilocus sequence-based approach has been emerged as a powerful tool to identify and type Bcc [18,21]. Thus, the present study aimed to identify the studied *Burkholderia* strains using multilocus sequence analysis and typing (MLSA-MLST), and to determine their biocontrol and plant growth promotion activity on four different Korean maize cultivars, namely Hikchal, Mibeak-2ho, Chahong-chal, and Oluckdehack-chal.

## 2. Results and Discussion 

In this study, the biochemical and genetic characteristics of two *Burkholderia* strains viz., KNU17BI2 and KNU17BI3, which were isolated from maize rhizosphere in South Korea, were investigated. 

### 2.1. Identification of Bacterial Isolates

**Figure 1:**
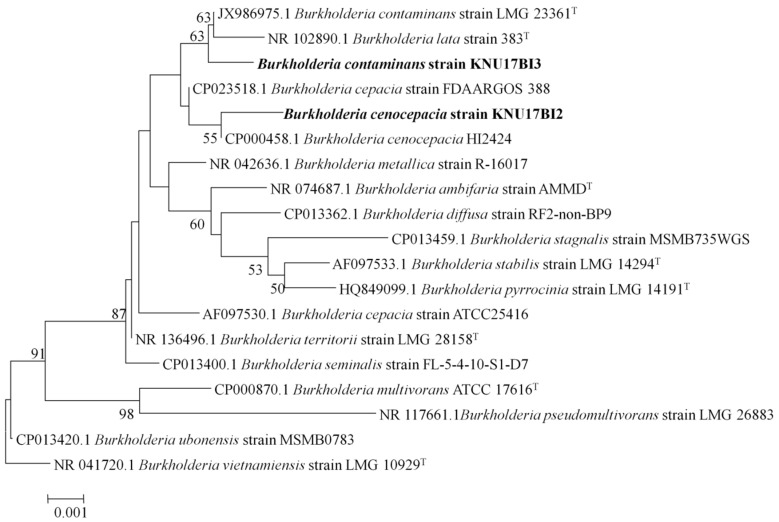
Neighbor-joining phylogenetic analysis based on the 16S rRNA gene sequences of *B. cenocepacia* strain KNU17BI2 and *B*. *contaminans* strain KNU17BI3. Bootstrap values were determined based on 1000 trials and bootstrap values only >50 are indicated at branch nodes. The scale bar represents the number of nucleotide substitutions per site. Sequences of type species are indicated by a symbol (T).

**Figure 2:**
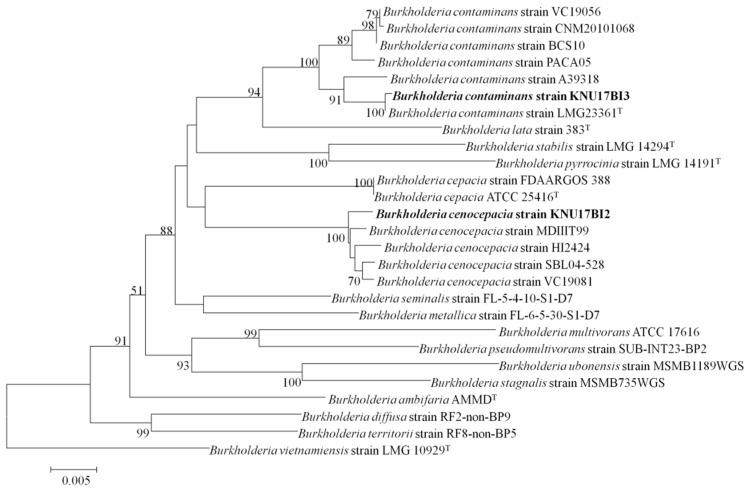
Phylogenetic tree based on MLSA of the concatenated nucleotide sequences of seven loci from the studied strains (*B. cenocepacia* strain KNU17BI2 and *B*. *contaminans* strain KNU17BI3) and reference strains of *Burkholderia* species. Values (>50%) that were based on 1000 bootstraps are shown at branch nodes. The scale bar represents the number of nucleotide substitutions per site. Sequences of type species are indicated by a symbol (T).

According to BLAST-based search, 16S rRNA gene sequence of the studied strains, KNU17BI2 and KNU17BI3, had high similarity (99%) to the 16S rRNA gene sequences of reference *Burkholderia* species (data not shown). The phylogenetic relationship based on 16S rRNA gene sequence analysis showed that strain KNU17BI2 and strain KNU17BI3 were closely related to *Burkholderia cenocepacia* and *Burkholderia contaminans* in our phylogenetic tree, respectively (Figure 1). Thus, for a better identification of the studied strains from other closely related to reference *Burkholderia* species, multilocus sequence analysis and typing (MLSA-MLST) was performed. MLSA-MLST is the best option for the identification of the *B. cepacia* complex [18,22,23]. Thus, the phylogenetic tree that is inferred from MLSA revealed that the strain KNU17BI2 and strain KNU17BI3 belong to *B. cenocepacia* and *B*. *contaminans*, respectively (Figure 2). More importantly, the result of multilocus sequence typing (MLST) analysis revealed that the strain KNU17BI2 belongs to *B. cenocepacia* was of a novel sequence type (ST) that was designated ST-1538. The novel ST-1538 differs from other closely related sequence types by at least three loci in the allelic profile (Table 1). On the other hand, strain KNU17BI3, identified as *B*. *contaminans*, had a similar allelic profile when compared to all of the seven loci of *B. contaminans* with the sequence type 102 of the strain LMG23361 (Table 1). The MLST data of our strain KNU17BI2 of sequence type 1538, including the nucleotide sequences of each loci, allelic profiles, as well as sequence type, have been deposited in the *Burkholderia cepacia* complex PubMLST database at http://pubmlst.org/bcc. The isolation of *B. cenocepacia* and *B. contaminans* from rhizospheric soil has previously been reported [14,24]. However, this is the first report on the isolation of *B. cenocepacia* in maize rhizosphere in South Korea. 

### 2.2. In Vitro Plant Growth Promoting (PGP) Activity Assays

**Figure 3:**
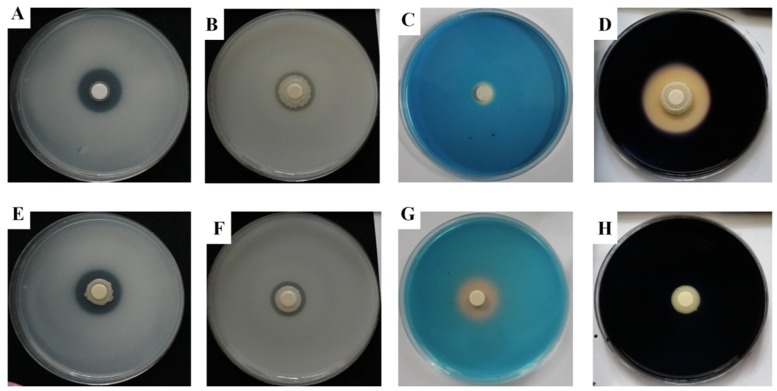
(**A**,**E**) In vitro phosphate solubilization on national botanical research institute’s phosphate growth (NBRIP) plates (**B**,**F**) zinc solubilization on Pikovskaya (PVK) agar plates supplemented with an insoluble ZnO (1.244 g/L) (**C**,**G**) siderophore production, and (**D**,**H**) amylase activity after seven days of incubation (top = strain KNU17BI2, bottom = strain KNU17BI3).

With regard to plant growth promoting traits, both of strains showed a promising effect. The results of phosphate solubilization assay revealed that the strains showed the ability to solubilize tricalcium phosphate (TCP) according to the formation of clear solubilization zone around its colony (Table 2 and Figure 3). Strain KNU17BI2 showed a higher phosphate solubilization index (SI) (2.3) as compared to strain KNU17BI3 (1.8) after 10 days of incubation. The phosphate solubilization index (SI) of both strains increased with the increasing incubation period (Table 2). The formation of the solubilized halo zone around the bacterial colonies may be due to the ability of the strains to produce phosphatase enzymes or other chemical compounds, such as organic acids and polysaccharides [25]. Similarly, the potentiality of *B. cenocepacia* to solubilize inorganic phosphates has been previously reported [26]. Phosphorus is the second most important nutrient after nitrogen for plant growth; however, only less than 5% of total soil phosphorus is found in available form to plants [27,28]. Thus, the ability to solubilize the insoluble phosphates is one of the most important features of plant growth promoting bacteria to enhance plant nutrition through an increase in phosphorus uptake by plants [29]. The application of phosphate solubilizing bacteria could contribute to the reduction of excessive fertilizers chemical usage and thereby reduce the harmful effects of fertilizers on the environment and human health [28].

In addition, the Zn solubilization assay result revealed that both strains had the potential to solubilize an insoluble zinc sources, ZnO (1.244 g·L^−1^) and ZnCO_3_, 1.913 g·L^−1^ in Pikovskaya (PVK) agar media. The Zn solubilization ability of strain KNU17BI2 was comparatively higher than KNU17BI3 on both tested zinc sources (Table 2 and Figure 3). In addition, both of the strains showed higher solubilization efficiency on ZnO amended PVK agar media than ZnCO_3_ media. These results comply with the previous studies [14,30] that *Burkholderia* species have the potential to solubilize the insoluble form of zinc. The solubilization potential may be attributed to the release of different chemical compounds, including gluconic acid and 2 keto gluconic acids [31]. Zn has the propensity to form insoluble complexes in the soil and become unavailable for plant uptake [32]. Thus, the lack of Zn is a continual challenge in crop production, being particularly in high pH soils, and its significant impact on human nutrition has been previously reported [33,34]. Interestingly, the solubilization of an insoluble soil Zn by bacterial inoculations has been reported to significantly increase in the total Zn uptake and thereby increase plant growth [32]. 

Both the studied strains developed yellow-orange halo around their colonies on blue Chrome Azurol S (CAS) agar medium signifying their ability to produce siderophore. Strain KNU17BI3 exhibited higher (23 mm) siderophore production, while KNU17BI2 exhibited very low siderophore (12 mm) after seven days of incubation (Figure 3). Similar to our study, previous studies [35,36] reported the potential of *Burkholderia* species to produce siderophores. A PGPR having siderophore production potential plays an important role in helping plants to acquire iron for optimal growth and it also offers competitive advantages to PGPR by suppressing the proliferation of plant pathogens [37,38].

**Figure 4:**
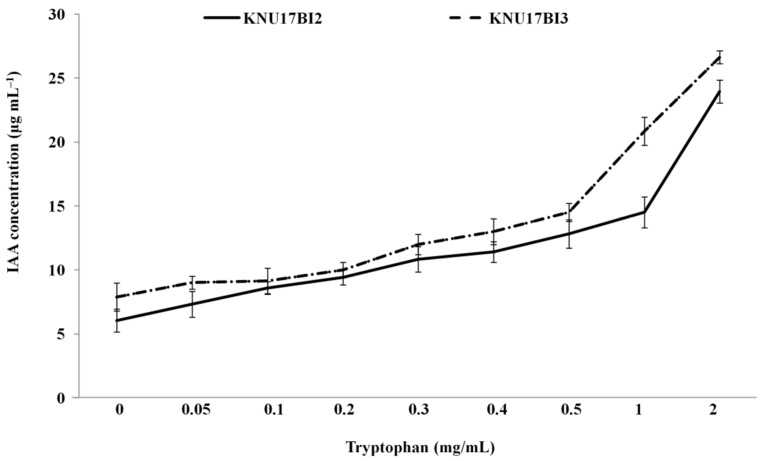
Effect of l-tryptophan concentration on indole acetic acid (IAA) production by strain KNU17BI2 and strain KNU17BI3 assessed after three days of incubation. The vertical bars indicate the standard error of three replications.

The studied strains displayed positive results for ammonia production and IAA production. The amount of ammonia that is produced by strain KNU17BI3 was by far higher (14.1 μg·mL^−1^) than strain KNU17BI2 (6.3 μg·mL^−1^) after three days of incubation. In addition, both of the strains were able to produce IAA in LB medium, regardless of amendment of L-tryptophan (Figure 4). After three days of incubation, the amount of IAA production by strain KNU17BI3 in LB medium without the amendment of l-tryptophan was higher (7.9 μg·mL^−1^) when compared to KNU17B2 (6.1 μg·mL^−1^) (Figure 4). In l-tryptophan amended LB medium, the amount of IAA was increased as L-tryptophan concentration increased in both strains (Figure 4). The maximum IAA (26.6 7.9 μg·mL^−1^) was produced by strain KNU17BI3 at higher l-tryptophan concentration (2.0 μg·mL^−1^). Indole-3-acetic acid (IAA) and ammonia are produced by many plant-associated bacteria that can enhance plant growth [39,40] with enhanced water and nutrients uptake [41]. 

The strains exhibited fast and luxuriant growth colonies on DF-agar plate that was supplemented with 3mM ACC as the unique nitrogen source (data not shown). This signifies ACC deaminase activity of the strains, as they were capable of using ACC as a nitrogen source. Our result complies with previous reports [42,43] that ACC deaminase is the widespread enzyme in diverse *Burkholderia* species, including *B. caryophylli, B. cenocepacia,* and *B. contaminnans*. Furthermore, strain KNU17BI2 exhibited a positive reaction for amylase, while strain KNU17BI3 did not show the activity (Figure 3). Amylase activity can help PGPR to easily hydrolyze the cell wall of phytopathogens [44].

### 2.3. In Vitro Antifungal Activity

**Figure 5:**
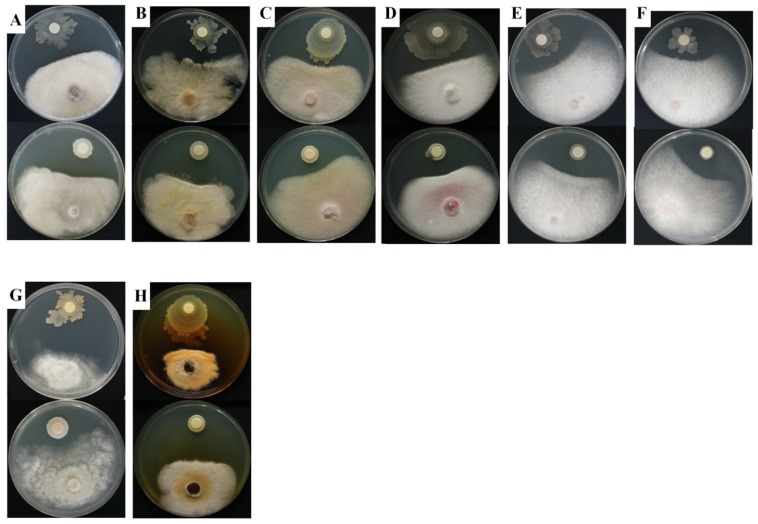
In vitro antifungal activity of the isolated *Burkholederia* strains against different phytopathogens in the dual culture: (**A**) *A. alternate*, (**B**) *F. graminearum*, (**C**) *F. moniliforme*, (**D**) *F. oxysporum* f.sp. *melonis*, (**E**) *F.* subglutinans, (**F**) *F. temperatum*, (**G**) *P. drechsleri*, and (**H**) *S. lycopersici* (top = *B. cenocepacia* strain KNUBI2, bottom = *B. contaminans* strain KNUBI3).

In the dual-culture assay, the antagonistic activity of strain KNU17BI2 and strain KNU17BI3 were tested against *F*. *temperatum*, maize root rot pathogen. The results revealed that both of the strains were effective in inhibiting the mycelial growth of *F*. *temperatum* (Table 3). The strains were further tested for their broad-spectrum activity against eight important phytopathogens (Table 3). The results showed that both of them had strong antifungal activity against all of the tested phytopathogens (Table 3 and Figure 5). The zone of inhibition around the tested plant pathogens (Figure 5) by the studied strains may be attributed to the ability of the strains to produce toxin metabolites, proteolytic enzymes, and siderophore [36,45]. Previous studies [14,15] reported the biocontrol potential of *Burkholderia* species, including *B. cepacia* and *B. contaminans* against several phytopathogens. However, the antifungal activity of *B. cenocepacia* against *Fusarium graminearum*, *Fusarium moniliforme*, *Fusarium oxysporum* f.sp. *melonis*, *Fusarium *subglutinans,* F*. *temperatum*, *Phytophthora drechsleri,* and *Stemphylium lycopersici* has not previously been reported. *F. graminearum,* F. *moniliforme,* and F. *subglutinans* are destructive disease of cereals that cause yield loss and grain quality due to their associated toxic metabolites [46,47]. *P. drechsleri* is one of the most devastating plant pathogen causing sever root rot in various crops [48,49]. *S. lycopersici* is an emerging plant pathogen causing leaf spot, stem rot, and fruit rot in various commercial crops [50,51,52]. This generally shows the essence of exploiting *B. cenocepacia* and *B. contaminans* for the control of the aforementioned pathogens, which causes huge economic loss in different crops.

### 2.4. SEM Analysis 

**Figure 6:**
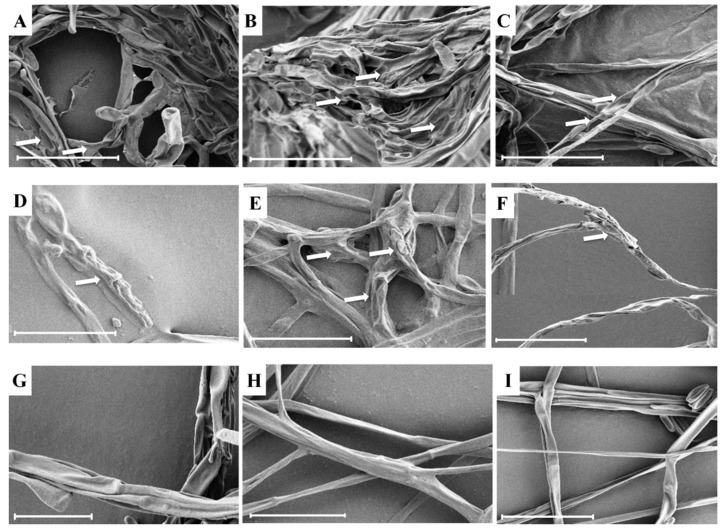
Scanning electron micrographs of (**A**,**D**,**G**) *F. moniliforme*, (**B**,**E**,**H**) *F. *subglutinans** and (**C**,**F**,**I**) *F*. *temperatum* (**A**–**C** = treated with strain KNU17BI2, **D**–**F** = treated with strain KNU17BI3, **G**–**I** = untreated control), (scale bars: 20 μm). (**A**) White arrows denote shrinked, deformed and/or lysed fungal structures.

Scanning electron microscope was used to examine any morphological defects from the edges of the inhibitory clear zone of the tested phytopathogens due to the studied strains. The SEM analysis revealed that the studied strains caused serious defects in the hyphal morphology of the selected phytopathogens, while hyphae from the untreated control were intact with normal morphology. The studied strains caused the hyphae of the phytopathogens to shrink, lyse, and deform (Figure 6). Similar to our study, such morphological defects in an interaction between bacterial strains and fungi in a dual culture have previously been reported [53]. However, to the best of our knowledge, the deleterious effects of *B. cenocepacia* against *F. moniliforme*, *F. *subglutinans, and* F*. *temperatum* have not previously been reported.

### 2.5. Greenhouse Experiments 

#### 2.5.1. Effect of Strain KNU17BI2 and Strain KNU17BI3 on Virulence of *F. temperatum*

**Figure 7:**
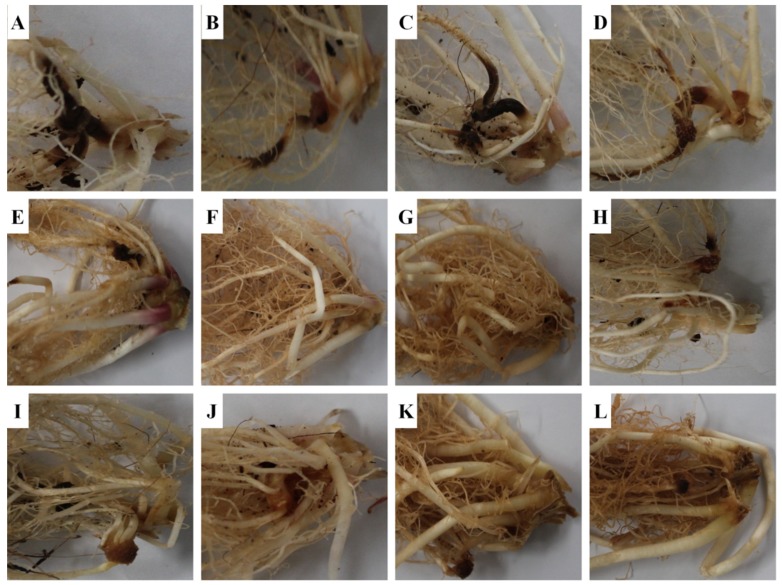
Pictorial view of roots of Korean maize cultivars infected with *F*. *temperatum* (**A**,**E**,**I**) Hikchal, (**B**,**F**,**J**) Mibeak-2ho, (**C**,**G**,**K**) Chahong-chal and (**D**,**H**,**L**) Oluckdehack-chal (**A**–**D** = negative control, **E**–**H** = treated with strain KNU17BI2, **I**–**L** = treated with strain KNU17BI3). Non-bacterized plants but pathogen-challenged were served as negative control.

**Figure 8:**
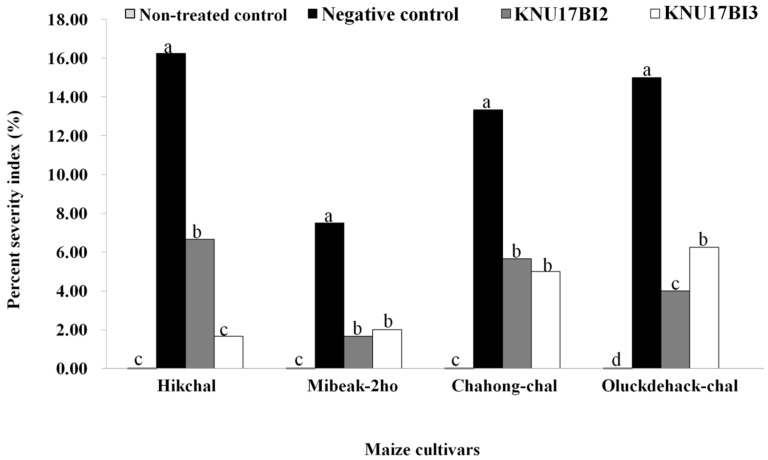
Effectiveness of strain KNU17BI2 and strain KNU17BI3 for the control of root rot of maize caused by *F*. *temperatum* in greenhouse. Percent severity index (mean of three replications having 10 plants per replication) assessed 30 days after treatment. Mean values having the same letter(s) in each cultivar are not statistically different (*p* ≤ 0.05) according to DMRT test. Non-treated plants (neither pathogen, nor *Burkholderia* strains) were served as non-treated control and non-bacterized plants, but pathogen-challenged were served as negative control.

The influence of KNU17BI2 and KNU17BI3 in reducing the virulence of *F. temperatum* on the seedlings of four maize cultivars viz., Hikchal, Mibeak-2ho, Chahong-chal, and Oluckdehack-chal was assessed after 30 days of planting. Subsequently, typical symptoms that were caused by the root rot pathogen, *F*. *temperatum,* were observed on pathogen-inoculated plants, as indicated by discolored primary and secondary roots [54,55] (Figure 7). The severity data showed that root rot disease was significantly reduced in all of the tested Korean maize cultivars following the soil drenching of the studied strains (Figure 8). To the contrary, non-bacterized plants, but pathogen-challenged (negative control treatment), had the highest *F*. *temperatum*-induced root rot severity (Figure 7 and Figure 8). The development of healthy roots on bacterized plants of each cultivar may be due to the direct effect of the *Burkholderia* strains on pathogen, *F. temperatum* [56]. Similar to our result, previous studies [7,57] reported the potential of PGPR to suppress maize root diseases. *Burkholderia cepacia* has been reported to suppress *Pythium* damping-off of sweet corn through lysis of zoospores and the prevention of cyst germination and germ tube growth [57]. Furthermore, Omar and his co-workers [58] demonstrated the bio-control capacity of *Burkholderia cepacia* against *Fusarium* crown and root rot of. However, no previous study has reported the potential of *B. cenocepacia* and *B. contaminans* to control root rot of maize that is caused by *F. temperatum*. Among, the four maize verities tested, root rot severity was comparatively higher on cv. Hikchal than cv. Mibeak-2ho (Figure 7 and Figure 8). This is the first report showing the reaction of Korean maize cultivars to the root rot causing pathogen, *F. temperatum.*

#### 2.5.2. Plant Growth Promotion Effect of Strain KNU17BI2 and Strain KNU17BI3 

**Figure 9:**
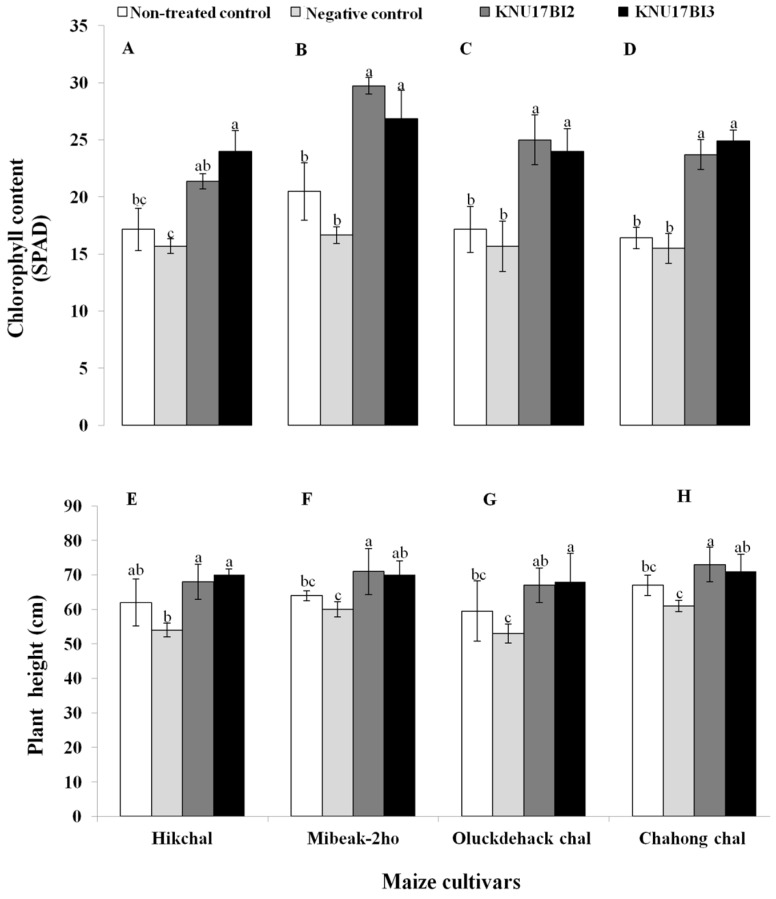
Effect of strain KNU17BI2 and strain KNU17BI3 on (**A**–**D**) total chlorophyll content and (**E**–**H**) plant height of Korean maize cultivars (left to right = Hikchal, Mibeak-2ho, Chahong-chal, and Oluckdehack-chal) after 30 days of planting. Mean values having the same letter(s) in each cultivar are not statistically different (*p* ≤ 0.05) according to the DMRT test. Non-treated plants (neither pathogen, nor *Burkholderia* strains) were served as non-treated control and non-bacterized plants, but pathogen-challenged were served as negative control.

**Figure 10:**
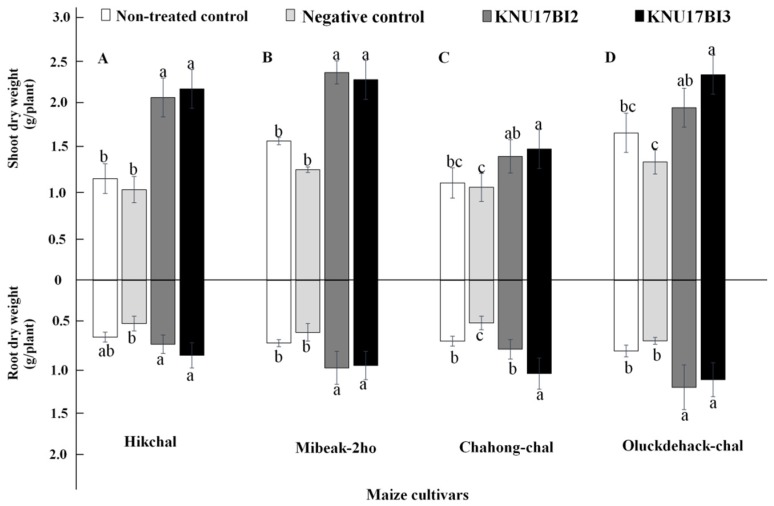
Effect of strain KNU17BI2 and KNU17BI3 on root and shoot biomass of Korean maize cultivars after 30 days of planting. Mean values having the same letter(s) in each cultivar are not statistically different (*p* ≤ 0.05) according to DMRT test. Non-treated plants (neither pathogen, nor *Burkholderia* strains) were served as non-treated control and non-bacterized plants, but pathogen-challenged were served as negative control.

The result of our study revealed that *F. temperatum* highly affected the negative control plants, thereby significantly reducing the shoot growth and amount of total chlorophyll content (Figure 9). On the other hand, plants that were treated with either of the strains had a highly significant positive effect on all plant growth parameters that were tested viz., total chlorophyll content, plant height, shoot dry weight, and root dry weight in all maize cultivars (Figure 9 and Figure 10). Similar to our study, previous studies [1,4] reported that seedling chlorosis, reduction in shoot growth, and fresh weight were observed on maize that was growing in *F. temperatum* inoculated soil. Furthermore, previous reports [1,59,60] explained that the degree of virulence by *F. temperatum* was defined as the inhibition of shoot elongation and chlorosis symptoms. More importantly, the increase in all tested plant growth parameters may be due to the plant growth promoting traits that the strains possess. In addition, the plant growth promotion effect of *Burkholderia* species on maize plant has been previously reported [14,61]. It is worth to note that *B. cenocepacia* has been reported as plant-beneficial endophytic bacterium to control *Fusarium* wilt of banana [62,63]. However, this is the first report demonstrating the bio-control potential of *B. cenocepacia* and *B. contaminans* against root rot of maize that is caused by *F*. *temperatum*.

In conclusion, MLSA-MLST revealed that strain KNU17BI2, which belongs to *B. cenocepacia,* was of a novel sequence type (ST) designated ST-1538. On the other hand, strain KNU17BI3, identified as *B*. *contaminans*, had similar allelic profile when compared to all seven loci of *B. contaminans* strain LMG 23361 with a sequence type 102. The current study reported two multi-trait bacterial strains viz., *B. cenocepacia* strain KNU17BI2 and *B. contaminans* strain KNU17BI3 possessing promising in vitro PGP traits, such as zinc solubilization, phosphate solubilization, ammonia production, 1-aminocyclopropane-1-carboxylate (ACC) deaminase activity, indole acetic acid (IAA) production, siderophore, and hydrolytic enzyme activity. This study is the first report on the strong in vitro antifungal activity of *B. cenocepacia* strain KNU17BI2 of novel ST against several economically important plant fungal pathogens. These include, against *F. temperatum*, *F. graminearum*, *F. moniliforme*, *F. oxysporum* f.sp. *melonis*, *F.* subglutinans, *P. drechsleri*, and *S. lycopersici*. In our study, root drenching of plants of four maize cultivars with the studied strains lead to an increase in the growth of the four maize cultivars and a significant positive effect in the control of root rot of maize seedlings on four different maize cultivars. The present research work reports, for the first time, the potential of *B. cenocepacia* and *B. contaminans* to control root rot of maize that is caused by *F. temperatum*. Hence, further studies are needed to precisely identify the prevailing bioactive chemical compounds behind such activities that would be novel sources of natural products for plant growth promotion as well as biological control. 

## 3. Materials and Methods 

### 3.1. Microbial Sources 

In this study, two bacterial strains (strain KNU17BI2 and strain KNU17BI3) were isolated from maize rhizosphere soil that was located in Gangwon-do province (37°86′ N, 127°75′ E), South Korea. For subsequent experiments, inoculum suspensions of each strain were grown in a tryptic soy broth (TSB) medium on a shaking incubator at 150 rpm in the dark at 28 ± 2 °C for 48 h. To harvest the bacterial cells, the growing media was *centrifuged* (6000 rpm, 5 min) and the bacterial cells were *washed* five times using phosphate-buffered saline solution (PBS; 5 mM K_2_HPO_4_, 150 mM NaCl, pH 7.0). Subsequently, the inoculum concentration of each strain was adjusted to 10^8^ cells·mL^−1^. 

The study phytopathogens were obtained from Korean agricultural culture collection (KACC), South Korea. The phytopathogens were: *A. alternate* (KACC43921), *F. graminearum* (KACC47499), *F. moniliforme* (KACC41032), *F. oxysporum* f.sp. *melonis* (KACC47669), *P. drechsleri* (KACC40190), and *S. lycopersici* (KACC40967). *F. temperatum* and *F. *subglutinans** were kindly provided by Prof. Kim Kyoung Su, Kangwon National University, South Korea. The tested phytopathogens were maintained on potato dextrose agar (PDA) plates at 4 °C for further experimental use and mycelia colonized PDA plugs (8 mm) of the pathogens from the edge of culture plate were used in all of the experiments. 

### 3.2. Identification of Bacterial Isolates

#### 16S rRNA Gene Sequencing

Almost the full-length 16S rRNA gene sequences were PCR amplified with the universal primers 27f (5′-AGAGTTTGATCATGGCTCAG-3′) and 1492R (5′-TACGGYTACCTTGTTACGACTT-3′) [64]. Sequencing was carried out at Macrogen Inc. (Seoul, South Korea) using a 3730XL DNA sequencer (Applied BioSystems, Waltham, CA, USA). The search for sequence similarity was carried out using the BLAST server (http://www.ncbi.nlm.nih.gov/BLAST/). The phylogenetic tree of 16S rRNA gene was constructed by the neighbor-joining method using Kimura’s two-parameter model [65] that was implemented in the MEGA 6 software [66]. A bootstrap confidence analysis was performed with bootstrap 1000 replicates. 

### 3.3. Multilocus Sequencing Analysis and Typing (MLSA-MLST) 

The studied strains were further studied by MLSA. In this analysis, the primers of the seven loci were used following the method of Spilker et al. [22]. The concatenated sequence of MLSA (2773 bp) were constructed as: *atp*D (443 bp), *glt*B (400 bp), *gyr*B (454 bp), *rec*A (393 bp), *lep*A (397 bp), *pha*C (385 bp), and *trp*B (301 bp). The concatenated sequences of each studied strain and the reference strain were used to construct the phylogenetic tree using MEGA6.0 [66]. For MLST, all seven housekeeping gene (*atp*D, *glt*B, *gyr*B, *rec*A, *lep*A, *pha*C and *trp*B) sequences of the studied strains were compared to reference strains available in the *Burkholderia cepacia* complex PubMLST database (https://pubmlst.org/bcc/). 

### 3.4. Genbank Accession Numbers

The nucleotide sequences of each allelic profiles and the sequence type of strain KNU17BI2 are found at the *Burkholderia cepacia* complex PubMLST database (http://pubmlst.org/bcc) with sequence type (ST) 1538. In addition, the nucleotide sequences of 16S rRNA gene and each MLSA-MLST loci of strain KNU17BI2 and strain KNU17BI3 have been deposited in GenBank/EMBL/DDBJ (Table 4).

### 3.5. In Vitro Antifungal Activity 

The broad-spectrum inhibitory activity of the studied strains was tested in vitro against seven different economically important plant fungal pathogens using dual culture technique on PDA. These were: *F. temperatum*, *A. alternate*, *F. graminearum*, *F. moniliforme*, *F. oxysporum* f.sp. *melonis*, *P. drechsleri*, and *S. lycopersici*. After five days of incubation at 28 ± 2 °C, the inhibitory activity of the strains was determined as the length of the zone of mycelial growth inhibition between the bacterial colony and the test phytopathogen. The percent inhibition of mycelial growth of the tested phytopathogens was calculated based on the formula: PI = (C − T)/C × 100, where PI is the inhibition of mycelial growth in percent, C is the radial growth of the phytopathogen in control, and T is the radial growth of the phytopathogen in dual culture. The experiment was carried out in three replications and the experiment was repeated three times. 

### 3.6. Scanning Electron Microscope (SEM) Analysis

To study the interaction of the selected phytopathogens (*F. moniliforme*, *F. *subglutinans, and* F*. *temperatum*) and the studied strains, the mycelia of the phytopathogens from the edge of the halo zone in the dual culture Petri plates were taken and then observed under scanning electron microscope (SEM). The mycelia samples were prepared by thin coating with gold and palladium (60:40). The coated mycelia samples were observed under SEM (LEO Model 1450VP Variable Pressure Scanning Electron Microscope; Carl Zeiss, Cambridge, MA, USA).

### 3.7. In Vitro Plant Growth Promoting (PGP) Activity Assays

The strains were also tested for important traits of plant growth promotion activity. The traits were: phosphate solubilization, zinc solubilization, indole-3-acetic acid (IAA) production, ammonia production, and siderophore production. For each trait, the experiments were conducted in triplicates and the experiment was repeated three times.

Phosphate solubilizing ability of the studied strains was assessed on national botanical research institute’s phosphate growth (NBRIP) medium that was supplemented with tricalcium phosphate (TCP). In addition, the zinc solubilizing potential of the strains was determined on PVK medium supplemented with an insoluble zinc sources containing 1% zinc (ZnO, 1.244 g·L^−1^ and ZnCO_3_, 1.913 g·L^−1^). After seven days of incubation at 28 ± 2 °C, the diameter of the clear zone around the bacterial colonies was measured. The solubilization index (SI) of phosphate and zinc by the studied strains was determined as the ratio between the halo zone diameter and the colony diameter. 

The potential of the studied strains to produce ammonia was determined following the method of Cappuccino and Sherman [67]. The amount of ammonia produced was spectrophotometrically determined (UV–1800, Shimadzu Corporation, Kyoto, Japan) from the standard curve of ammonium sulfate (0–10 µmol·mL^−1^). The method of Gordon and Weber [68] was employed to determine the potential of the tested strains to produce indole acetic acid (IAA). The concentration of IAA in culture was determined while using a standard curve of IAA that was prepared by diluting pure IAA (Sigma-Aldrich, St. Loise, MO, USA) in LB medium at various concentrations (0 to 2 mg·mL^−1^). The strains were also qualitatively tested for the aminocyclopropane-1-carboxylate (ACC) deaminase activity on Dworkin and Foster (DF) minimal salt medium amended with 3 mM filter sterilized ACC as the sole nitrogen source [69]. Amylase activity was determined on starch agar plates [67].

### 3.8. Greenhouse Experiments 

Strain KNU17BI2 and strain KNU17BI3 were further evaluated for biological control of maize root rot on four Korean maize cultivars under greenhouse conditions. The cultivars were Hikchal, Mibeak-2ho, Chahong-chal, and Oluckdehack-chal. In this study, four mycelia colonized PDA plugs (8 mm) of *F. temperatum* were mixed with the top 5 cm of soil in each plastic pot (10 cm diameter) [70]. After 24 h of pathogen inoculation, the seeds of each cultivar were planted at a depth of approximately 4 cm (one seed per pot). Subsequently, one milliliter of bacterial inoculum adjusted at a concentration of 10^8^ cells·mL^−1^ was applied to seeds of each cultivar prior to covering with soil. Non-treated plants (neither pathogen, nor *Burkholderia* strains) were served as non-treated control and non-bacterized plants, but the pathogen-challenged were served as negative control. In this experiment, treatments (single *Burkholderia* strains, non-treated control, and negative control) were arranged in a completely randomized design (CRD) with three replications with 10 plants per replication, and the experiment was repeated twice. 

### 3.9. Assessment of Effect of Burkholderia Strains on Virulence of F. temperatum

The effect of strain KNU17BI2 and strain KNU17BI3 in controlling the root rot of maize due to *F. temperatum* on the four Korean maize cultivars was assessed after 30 days of planting. Disease scaling of root rot that is caused by *F. temperatum* was done based on a 0–4 scale, where 0 = 0, healthy root; 1, less than 25% of the root spoiled due to rotting; 2, 25–50% of the root spoiled, evident from dropping of the leaves during daytime; 3, up to 75% of the root damaged, as evident from starting of wilt and drying of leaves from bottom to top; and, 4, complete rotting of the root, completely wilted, dead, and dry plants [57]. For analysis, the scale was converted into the percentage severity index (PSI) [71].

$$\mathrm{PSI}=\frac{\sum \;\mathrm{of}\;\mathrm{all}\;\mathrm{numerical}\;\mathrm{ratings}\;\times \;100}{\mathrm{Total}\;\mathrm{number}\;\mathrm{of}\;\mathrm{observations}\;\times \;\mathrm{maximum}\;\mathrm{score}\;\mathrm{on}\;\mathrm{scale}}$$

Furthermore, based on previous studies [37,38], the degree of virulence that is caused by *F. temperatum* was defined as the inhibition of shoot elongation and chlorosis symptoms. Hence, leaf chlorosis that was based on total chlorophyll content (SPAD unit) was measured using chlorophyll-meter SPAD 502 (Konica Minolta, Tokyo, Japan) from the middle leaf of each plant. Morphological data, including shoot and root dry weight (oven dried at 65 °C for 72 h), were also recorded. 

### 3.10. Statistical Data Analysis

Analysis of variance (ANOVA) of all experimental data was carried out using SAS software version 9.2 [72]. All of the experiments of in vitro and greenhouse were conducted in three replications and were repeated twice. The results of the experiments were expressed as mean ± standard error. Mean separation was carried out using the multiple comparison procedure and Duncan’s multiple range test (DMRT) at *p* ≤ 0.05.

## Figures and Tables

**Table 1:** Allelic profile of the seven loci of strain KNU17BI2 and strain KNU17BI3 as compared with closely related strains of *B*. *cenocepacia* and *B*. *contaminans*.

Strain	Species	Source	MLST ^a^							
			atpD	gltB	gyrB	recA	lepA	phaC	trpB	ST
**KNU17BI2**	***B. cenocepacia***	**Environmental**	**23**	**605**	**307**	**15**	**93**	**8**	**144**	**1538**
HI2424	*B. cenocepacia*	Environmental	23	134	57	15	93	8	14	122
MDIII-T99	*B. cenocepacia*	Environmental	23	16	86	15	93	8	14	125
SBL04-528	*B. cenocepacia*	Clinical	23	16	352	15	93	6	14	414
VC19081	*B. cenocepacia*	Clinical	16	134	352	15	93	8	144	1500
**KNU17BI3**	***B. contaminans***	**Environmental**	**64**	**80**	**76**	**89**	**105**	**97**	**70**	**102**
LMG 23361	*B. contaminans*	Environmental	64	80	76	89	105	97	70	102
VC19056	*B. contaminans*	Clinical	151	192	245	152	158	173	151	482
PACA05	*B. contaminans*	Clinical	151	192	245	152	11	173	151	637
BCS10	*B. contaminans*	Clinical	64	192	245	152	158	173	151	716
CNM20101068	*B. contaminans*	Clinical	318	192	245	152	158	173	151	771
A39318	*B. contaminans*	NA	64	192	631	378	428	118	420	912

^a^ Multilocus sequence type (ST). NA—not available.

**Table 2:** Qualitative estimation of phosphate and zinc solubilization efficiency of the studied strains.

Solubilization	Incubation Period (Days)	Strains			
		KNU17BI2		KNU17BI3	
		Halo Zone Diameter (mm) ^a^	Solubilization Index	Halo Zone Diameter (mm) ^a^	Solubilization Index
**Phosphate**	7	17.0 ± 1.15	1.9	23.0±2.25	1.6
	10	21.5±1.21	2.3	30.0±1.20	1.8
**ZnO ^b^**	7	21.6±0.50	1.2	19.0±1.58	1.4
	10	25.2±1.34	1.3	22.4±2.2	1.5
**ZnCO_3_^b^**	7	17.0±1.80	1.1	18.0±1.28	1.2
	10	19.6±1.60	1.2	21.8±1.29	1.3

^a^ Values are means of three replications; ^b^ Insoluble zinc sources containing 1% zinc (ZnO, 1.244 g·L^−1^ and ZnCO_3_, 1.913 g·L^−1^).

**Table 3:** Broad spectrum antifungal activity of the study *Burkholederia* strains.

Target Phytopathogens	Percent Inhibition Zone (mm) ^a^ (Mean ± SE)	
	KNU17BI2	KNU17BI3
*A. alternate*	44.4 ± 1.5 ^b,c^	46.7 ± 1.4 ^a,b,c^
*F. graminearum*	44.0 ± 2.2 ^b,c^	50.0 ± 1.5 ^a,b^
*F. moniliforme*	50.1 ± 1.5 ^a,b,c^	41.1 ± 0.8 ^c,d^
*F. oxysporum* f.sp. *melonis*	50.2 ± 0.9 ^a,b,c^	47.8 ± 1.2 ^a,b^
*F. subglutinans*	44.5 ± 0.9 ^b,c^	41.0 ± 0.9 ^c,d^
*F. temperatum*	41.2 ± 0.9 ^c^	40.0 ± 0.9 ^d^
*P. drechsleri*	58.9 ± 2.1 ^a^	38.9 ± 2.5 ^d^
*S. lycopersici*	55.6 ± 2.3 ^a,b^	51.6 ± 1.8 ^a^

^a^ Values are means of three replications. Mean values having the same letter(s) in each a column are not statistically different (*p* ≤ 0.05) according to the Duncan’s multiple range test (DMRT) test.

**Table 4:** Genebank accession numbers of strain KNU17BI2 and KNU17BI3.

Loci	Accession Number	
	KNU17BI2	KNU17BI3
16S rDNA	MK212365	MK212366
*atp*D	MK225579	MK225586
*glt*B	MK225580	MK225587
*gyr*B	MK225581	MK225588
*lep*A	MK225582	MK225589
*pha*C	MK225583	MK225590
*rec*A	MK225584	MK225591
*trp*B	MK225585	MK225592

## References

[1] Varela C.P., Casal O.A., Padin M.C., Martinez V.F., Oses M.S., Scauflaire J., Munaut F., Castro M.B., Vázquez J.M. (2013). First report of *Fusarium temperatum* causing seedling blight and stalk rot on maize in Spain. Plant Dis..

[2] Czembor E., Stępień Ł., Waśkiewicz A. (2014). *Fusarium temperatum* as a new species causing ear rot on maize in Poland. Plant Dis..

[3] Shin J.H., Han J.H., Lee J.K., Kim K.S. (2014). Characterization of the maize stalk rot pathogens *Fusarium subglutinans* and *F. temperatum* and the effect of fungicides on their mycelial growth and colony formation. Plant Pathol. J..

[4] Lanza F.E., Mayfield D.A., Munkvold G.P. (2016). First report of *Fusarium temperatum* causing maize seedling blight and seed rot in North America. Plant Dis..

[5] Wang J.H., Zhang J.B., Li H.P., Gong A.D., Xue S., Agboola R.S., Liao Y.C. (2014). Molecular identification, mycotoxin production and comparative pathogenicity of *Fusarium temperatum* isolated from maize in China. J. Phytopathol..

[6] Reid T.C., Hausbeck M.K., Kizilkaya K. (2002). Use of fungicides and biological controls in the suppression of *Fusarium* crown and root rot of asparagus under greenhouse and growth chamber conditions. Plant Dis..

[7] Figueroa-López A.M., Cordero-Ramírez J.D., Martínez-Álvarez J.C., López-Meyer M., Lizárraga-Sánchez G.J., Félix-Gastélum R., Castro-Martínez C., Maldonado-Mendoza I.E. (2016). Rhizospheric bacteria of maize with potential for biocontrol of *Fusarium verticillioides*. SpringerPlus.

[8] Vejan P., Abdullah R., Khadiran T., Ismail S., Nasrulhaq Boyce A. (2016). Role of plant growth promoting rhizobacteria in agricultural sustainability—A review. Molecules.

[9] Shrivastava P., Kumar R. (2015). Soil salinity: A serious environmental issue and plant growth promoting bacteria as one of the tools for its alleviation. Saudi J. Biol. Sci..

[10] Coenye T., Vandamme P. (2003). Diversity and significance of *Burkholderia* species occupying diverse ecological niches. Environ. Microbiol..

[11] Gouda S., Das G., Sen S.K., Shin H.S., Patra J.K. (2016). Endophytes: A treasure house of bioactive compounds of medicinal importance. Front. Microbial..

[12] Lee J.W., Kim Y.E., Park S.J. (2018). *Burkholderia* alba sp. nov., isolated from a soil sample on Halla mountain in Jeju island. J. Microbiol..

[13] Carvalho-Gonçalves L.C., Gorlach-Lira K. (2018). Lipases and biosurfactants production by the newly isolated *Burkholderia* sp.. Braz. J. Biol. Sci..

[14] Tagele S.B., Kim S.W., Lee H.G., Kim H.S., Lee Y.S. (2018). Effectiveness of multi-trait *Burkholderia contaminans* KNU17BI1 in growth promotion and management of banded leaf and sheath blight in maize seedling. Microbiol. Res..

[15] Zhao K., Penttinen P., Zhang X., Ao X., Liu M., Yu X., Chen Q. (2014). Maize rhizosphere in Sichuan, China, hosts plant growth promoting *Burkholderia cepacia* with phosphate solubilizing and antifungal abilities. Microbiol. Res..

[16] Esmaeel Q., Pupin M., Jacques P., Leclère V. (2018). Nonribosomal peptides and polyketides of *Burkholderia*: New compounds potentially implicated in biocontrol and pharmaceuticals. Environ. Sci. Pollut. Res..

[17] Kandel S.L., Firrincieli A., Joubert P.M., Okubara P.A., Leston N.D., McGeorge K.M. (2017). An in vitro study of bio-control and plant growth promotion potential of *Salicaceae* endophytes. Front. Microbiol..

[18] Vandamme P., Dawyndt P. (2011). Classification and identification of the *Burkholderia cepacia* complex: Past, present and future. Syst. Appl. Microbiol..

[19] Yabuuchi E., Kosako Y., Oyaizu H., Yano I., Hotta H., Hashimoto Y., Ezaki T., Arakawa M. (1992). Proposal of *Burkholderia* gen. nov. and transfer of seven species of genus *Pseudomonas* homology group II to the new genus, with the type species *Burkholderia cepacia* (Palleroni and Holmes, 1981) comb. nov.. Microbiol. Immunol..

[20] Estrada-de los Santos P., Palmer M., Chávez-Ramírez B., Beukes C., Steenkamp E., Briscoe L. (2018). Whole Genome Analyses Suggests that *Burkholderia* sensu lato Contains Two Additional Novel Genera (*Mycetohabitans* gen. nov., and *Trinickia* gen. nov.): Implications for the Evolution of Diazotrophy and Nodulation in the *Burkholderia*ceae. Genes.

[21] Coenye T., Vandamme P., Govan J.R., LiPuma J.J. (2001). Taxonomy and identification of the *Burkholderia cepacia* complex. J. Clin. Microbiol..

[22] Spilker T., Baldwin A., Bumford A., Dowson C.G., Mahenthiralingam E., LiPuma J.J. (2009). Expanded multilocus sequence typing for *Burkholderia* species. J. Clin. Microbiol..

[23] Fila L., Dřevínek P. (2017). *Burkholderia cepacia* complex in cystic fibrosis in the post-epidemic period: Multilocus sequence typing-based approach. Folia Microbiol..

[24] Yoder-Himes D.R., Chain P.S.G., Zhu Y., Wurtzel O., Rubin E.M., Tiedje J.M., Sorek R. (2009). Mapping the *Burkholderia cenocepacia* niche response via high-throughput sequencing. Proc. Natl. Acad. Sci. USA.

[25] Goenadi D.H., Sisweto I., Sugiarto Y. (2000). Bioactivation of poorly soluble phosphate rocks with a phosphorus- solubilizing fungus. Soil Sci. Soc. Am. J..

[26] Mahamuni S.V. (2015). *Anticeratocystis paradoxa* and *Alternaria alternata* attribute of *Burkholderia cenocepacia* strain vimp 01 (jq867371). Int. J. Bioassays.

[27] Otieno N., Lally R.D., Kiwanuka S., Lloyd A., Ryan D., Germaine K.J., Dowling D.N. (2015). Plant growth promotion induced by phosphate solubilizing endophytic *Pseudomonas* isolates. Front. Microbiol..

[28] Shahid M., Hameed S., Tariq M., Zafar M., Ali A., Ahmad N. (2015). Characterization of mineral phosphate-solubilizing bacteria for enhanced sunflower growth and yield-attributing traits. Ann. Microbiol..

[29] Taurian T., Anzuay M.S., Angelini J.G., Tonelli M.L., Ludueña L., Pena D., Ibáñez F., Fabra A. (2010). Phosphate-solubilizing peanut associated bacteria: Screening for plant growth-promoting activities. Plant Soil.

[30] Dinesha R., Srinivasana V., Hamza S., Sarathambal C., Ankegowda S.J., Ganeshamurthy A.N., Gupta S.B., Nair V.A., Subila K.P., Lijina A., Divya V.C. (2018). Isolation and characterization of potential Zn solubilizing bacteria from soil and its effects on soil Zn release rates, soil available Zn and plant Zn content. Geoderma.

[31] Cozzi D., Desidevi P.G., Lepri L. (1969). The mechanism of ion exchange with algenic acid. J. Chromatogr..

[32] Vaid S.K., Kumar B., Sharma A., Shukla A.K., Srivastava P.C. (2014). Effect of Zn solubilizing bacteria on growth promotion and Zn nutrition of rice. J. Soil Sci. Plant Nutr..

[33] Johnson-Beebout S.E., Lauren J.G., Duxbury J.M. (2009). Immobilization of zinc fertilizer in flooded soils monitored by adapted DTPA soil test. Commun. Soil Sci. Plant Anal..

[34] Natheer S.E., Muthukkaruppan S. (2012). Assessing the in vitro zinc solubilization potential and improving sugarcane growth by inoculating *Gluconacetobacter diazotrophicus*. Ann. Microbiol..

[35] Ong K.S., Lee L.H., Yule C.M., Cheow Y.L., Lee S.M. (2016). *Burkholderia paludis* sp. nov. an antibiotic-siderophore producing novel *Burkholderia cepacia* Complex Species, isolated from Malaysian tropical peat swamp soil. Front. Microbiol..

[36] Deng P., Wang X., Baird S.M., Showmaker K.C., Smith L., Peterson D.G., Lu S. (2016). Comparative genome-wide analysis reveals that *Burkholderia contaminans* MS14 possesses multiple antimicrobial biosynthesis genes but not major genetic loci required for pathogenesis. MicrobiologyOpen.

[37] Hrynkiewicz K., Baum C., Leinweber P. (2010). Density, metabolic activity and identity of cultivable rhizosphere bacteria on *Salix viminalis* in disturbed arable and landfill soils. J. Plant Nutr. Soil Sci..

[38] Haas D., Défago G. (2005). Biological control of soil-borne pathogens by fluorescent pseudomonads. Nat. Rev. Microbiol..

[39] Vacheron J., Desbrosses G., Bouffaud M.L., Touraine B., Moënne-Loccoz Y., Muller D. (2013). Plant growth-promoting rhizobacteria and root system functioning. Front. Plant Sci..

[40] Khan A.L., Gilani S.A., Waqas M., Al-Hosni K., Al-Khiziri S., Kim Y.H. (2017). Endophytes from medicinal plants and their potential for producing indole acetic acid, improving seed germination and mitigating oxidative stress. J. Zhejiang Univ.-SC B.

[41] Collavino M.M., Sansberro P.A., Mroginski L.A., Aguilar O.M. (2010). Comparison of in vitro solubilization activity of diverse phosphate-solubilizing bacteria native to acid soil and their ability to promote *Phaseolus vulgaris* growth. Biol. Fertil. Soils.

[42] Onofre-Lemus J., Hernandez-Lucas I., Girard L., Caballero-Mellado J. (2009). ACC (1-Aminocyclopropane-1-Carboxylate) deaminase activity, a widespread trait in *Burkholderia* species, and its growth-promoting effect on tomato plants. Appl. Environ. Microbiol..

[43] Singh R.P., Shelke G.M., Kumar A., Jha P.N. (2015). Biochemistry and genetics of ACC deaminase: A weapon to “stress ethylene” produced in plants. Front. Microbiol..

[44] Karthik C., Elangovan N., Kumar T.S., Govindharaju S., Barathi S., Oves M., Arulselvi P.I. (2017). Characterization of multifarious plant growth promoting traits of rhizobacterial strain AR6 under Chromium (VI) stress. Microbiol. Res..

[45] Hu Q.P., Xu J.G., Song P., Song J.N., Chen W.L. (2008). Isolation and identification of a potential biocontrol agent *Bacillus subtilis* QM3 from Qinghai yak dung in China. World J. Microbiol. Biotechnol..

[46] Ncube E., Flett B.C., Waalwijk C., Viljoen A. (2011). *Fusarium* spp. and levels of fumonisins in maize produced by subsistence farmers in South Africa. S. Afr. J. Sci..

[47] Matarese F., Sarrocco S., Gruber S., Seidl-Seiboth V., Vannacci G. (2012). Biocontrol of *Fusarium* head blight: Interactions between *Trichoderma* and mycotoxigenic *Fusarium*. Microbiology.

[48] Axel C., Zannini E., Coffey A., Guo D.M., Arendt E.K. (2012). Ecofriendly control of potato late blight causative agent and the potential role of lactic acid bacteria: A review. Appl. Microbiol. Biotechnol..

[49] Maleki M., Mokhtarnejad L., Mostafaee S. (2011). Screening of rhizobacteria for biological control of cucumber root and crown rot caused by *Phytophthora drechsleri*. Plant Pathol. J..

[50] 50Tomioka K., Sato T. (2011). Fruit rot of sweet pepper caused by *Stemphylium lycopersici* in Japan. J. Gen. Plant Pathol..

[51] Kurose D., Hoang L.H., Furuya N., Takeshita M., Sato T., Tsushima S., Tsuchiya K. (2014). Pathogenicity of *Stemphylium lycopersici* isolated from rotted tobacco seeds on seedlings and leaves. J. Gen. Plant Pathol..

[52] Nasehi A., Kadir J., Nasr-Esfahani M., Abed-Ashtiani F., Golkhandan E., Ashkani S. (2016). Identification of the new pathogen (*Stemphylium lycopersici*) causing leaf spot on Pepino (*Solanum muricatum*). J. Phytopathol..

[53] Torres M.J., Brandan C.P., Sabaté D.C., Petroselli G., Erra-Balsells R., Audisio M.C. (2017). Biological activity of the lipopeptide-producing *Bacillus amyloliquefaciens* PGPBacCA1 on common bean *Phaseolus vulgaris* L. pathogens. Biol. Control.

[54] Soonthornpoct P., Trevathan L.E., Ingram D. (2000). The colonization of maize seedling roots and rhizosphere by *Fusarium* spp. in Mississippi in two soil types under conventional tillage and notillage systems. Phytoprotection.

[55] Robles-Barrios K.F., Medina-Canales M.G., Rodríguez-Tovar A.V., Pérez N.O. (2015). Morphological and molecular characterization, enzyme production and pathogenesis of *Fusarium temperatum* on corn in Mexico. Can. J. Plant Pathol..

[56] Pal K.K., Tilak K.V.B.R., Saxcna A.K., Dey R., Singh C.S. (2001). Suppression of maize root diseases caused by *Macrophomina phaseolina*, *Fusarium moniliforme* and *Fusarium graminearum* by plant growth promoting rhizobacteria. Microbiol. Res..

[57] Heungens K., Parke J.L. (2000). Zoospore homing and infection events: Effects of the biocontrol bacterium *Burkholderia cepacia* AMMDR1 on two oomycete pathogens of pea (*Pisum sativum* L.). Appl. Environ. Microb..

[58] Omar I., O’neill T.M., Rossall S. (2006). Biological control of *Fusarium* crown and root rot of tomato with antagonistic bacteria and integrated control when combined with the fungicide carbendazim. Plant Pathol..

[59] Desjardins A.E., Plattner R.D., Nelsen T.C., Leslie J.F. (1995). Genetic analysis of fumonisin production and virulence of *Gibberella fujikuroi* mating population A (*Fusarium moniliforme*) on maize (*Zea mays*) seedlings. Appl. Environ. Microbiol..

[60] Scauflaire J., Gourgue M., Callebaut A., Munaut F. (2012). *Fusarium temperatum*, a mycotoxin-producing pathogen of maize. Eur. J. Plant Pathol..

[61] Kifle M.H., Laing M.D. (2016). Isolation and screening of bacteria for their diazotrophic potential and their influence on growth promotion of maize seedlings in greenhouses. Front. Plant Sci..

[62] Ho Y.N., Chiang H.M., Chao C.P., Su C.C., Hsu H.F., Guo C.T. (2015). In planta biocontrol of soilborne *Fusarium* wilt of banana through a plant endophytic bacterium, *Burkholderia cenocepacia* 869T2. Plant Soil.

[63] Ho Y.N., Huang C.C. (2015). Draft genome sequence of *Burkholderia cenocepacia* strain 869T2; a plant-beneficial endophytic bacterium. Genome Announc..

[64] Lane D.J., Stackebrandt E., Goodfellow M. (1991). 16S/23S rRNA sequencing. Nucleic Acid Techniques in Bacterial Systematics.

[65] Kimura M. (1980). A simple method for estimating evolutionary rate of base substitution through comparative studies of nucleotide sequences. J. Mol. Evol..

[66] Tamura K., Stecher G., Peterson D., Filipski A., Kumar S. (2013). MEGA6: Molecular evolutionary genetics analysis version 6.0. Mol. Biol. Evol..

[67] Cappuccino J.G., Sherman N. (1996). Microbiology: A Laboratory Manual.

[68] Gordon S.A., Weber R.P. (1951). Colorimetric estimation of indoleacetic acid. Plant Physiol..

[69] Naik P.R., Sahoo N., Goswami D., Ayyadurai N., Sakthivel N. (2015). Genetic and functional diversity among fluorescent psedumonas isolated from the rhizosphere of banana. Microb. Ecol..

[70] Liu K., Newman M., McInroy J.A., Hu C.H., Kloepper J.W. (2017). Selection and assessment of plant growth-promoting rhizobacteria for biological control of multiple plant diseases. Phytopathology.

[71] Wheeler B.E.J. (1969). An Introduction to Plant Diseases.

[72] SAS Institute Inc. (2008). SAS/STAT^®^ 9.2 User’s Guide.

